# Sponge-associated bacteria mineralize arsenic and barium on intracellular vesicles

**DOI:** 10.1038/ncomms14393

**Published:** 2017-02-24

**Authors:** Ray Keren, Boaz Mayzel, Adi Lavy, Iryna Polishchuk, Davide Levy, Sirine C. Fakra, Boaz Pokroy, Micha Ilan

**Affiliations:** 1 Department of Zoology, George S. Wise Faculty of Life Sciences, Tel Aviv University, Tel Aviv 69978, Israel; 2 Faculty of Materials Engineering and the Russell Berrie Nanotechnology Institute, Technion, Israel Institute of Technology, Haifa 32000, Israel; 3 Advanced Light Source, Lawrence Berkeley National Lab, Berkeley, California 94720, USA

## Abstract

Arsenic and barium are ubiquitous environmental toxins that accumulate in higher trophic-level organisms. Whereas metazoans have detoxifying organs to cope with toxic metals, sponges lack organs but harbour a symbiotic microbiome performing various functions. Here we examine the potential roles of microorganisms in arsenic and barium cycles in the sponge *Theonella swinhoei*, known to accumulate high levels of these metals. We show that a single sponge symbiotic bacterium, *Entotheonella* sp., constitutes the arsenic- and barium-accumulating entity within the host. These bacteria mineralize both arsenic and barium on intracellular vesicles. Our results indicate that *Entotheonella* sp. may act as a detoxifying organ for its host.

## Abstract (web summary)

The marine sponge *Theonella swinhoei* accumulates toxic arsenic and barium. Here the authors show that these toxic elements are actually accumulated and mineralized within vesicles inside bacteria that live within the sponge tissues.

Microorganisms greatly influence arsenic^1^ and barium^2^ geochemical cycles. Of the two, arsenic is extremely toxic to most life forms, except for a few arsenic-respiring bacteria^1^ and eukaryotic hyperaccumulators^3^. Most soluble forms of arsenic^3^, as well as the barium ion^4^, are potent bioavailable toxins. However, their bioavailability decreases when assimilated into organic forms, or by mineralization^4^,^5^.

Soluble barium concentrations in surface seawater are typically 10 μg l^−1^ and present in two states: most is found as a divalent barium ion and a small amount as undissociated barite (BaSO_4_)^6^. Barite is primarily detected as particulate matter and is especially enriched in areas of high microplankton productivity^6^. Arsenic is a common trace element (∼2 μg l^−1^) in marine environments. In the oxygenated seawater where *Theonella swinhoei* is found, arsenate is the dominant form of arsenic, followed by arsenite and a minute amount of methylated arsenic^3^. Most marine organisms accumulate arsenic to some extent, usually as organoarsenicals^3^. Although both arsenic and barium can be detected in all tissues, their distribution is not homogenous. Toxic element concentration is highest in tissues and organs dedicated to detoxification and excretion^3^,^7^.

Sponges are ancient metazoans^8^, of paramount significance to benthic communities^9^. Inhabiting all types of marine ecosystems, sponges contribute to global carbon^10^, nitrogen^11^ and silicon^12^ cycles. Sponges are filter feeders, filtering seawater up to 50,000 times their body volume daily^13^, resulting in high exposure to trace elements. Several studies have shown that sponges have the tendency, differing by species, to accumulate trace elements^14^,^15^,^16^,^17^. Sponge-associated bacteria are known to contribute to important elemental cycling in sponges, namely carbon, nitrogen and sulfur^8^, but their role in trace element cycling is rarely studied^18^,^19^.

Recent trace element analyses of Red Sea (Gulf of Aqaba) demosponges singled out *Theonella swinhoei*, a common Indo-Pacific sponge^20^, as a hyperaccumulator of arsenic (As) and barium (Ba)^14^. The concentrations of arsenic (8,600 μg g^−1^) and barium (13,400 μg g^−1^) are the highest concentrations recorded in any organism from an uncontaminated environment.

Symbiotic bacteria comprise approximately half the volume of *T. swinhoei*^21^. These include both phototrophic and heterotrophic bacteria^21^. The phototrophic cyanobacteria are located in a thin layer, close to the surface of the sponge, giving the host its characteristic colour. The heterotrophic bacteria reside mostly in the sponge's inner mesohyl part. While the majority of sponge-associated bacteria are unicellular^21^, the most abundant bacterium is filamentous and identified as *Entotheonella* sp.^21^,^22^. Moreover, *Entotheonella* sp. is considered a ‘talented producer', synthesizing many of the bioactive compounds found in this sponge^22^.

Because sponges lack organs and tissues, there is no apparent localization for the storage of arsenic and barium. Following the initial discovery of arsenic and barium accumulation, the sponge was separated into enriched fractions of either sponge cells or bacterial cells, revealing arsenic was localized to the bacterial fraction. Barium concentrations were also shown to be high in these fractions (although the differences were not statistically significant)^18^. Culturing trials recovered numerous arsenic-tolerant, sponge-associated bacteria growing on media enriched with as much as 100 mM arsenate^18^.

Here we show that a population of a single symbiotic bacterium, *Entotheonella* sp., appears to drive arsenic and barium accumulation in this sponge. We further demonstrate that the bacterium can mineralize both elements on intracellular membrane vesicles.

## Results

### *Entotheonella* sp. accumulates arsenic and barium

**Figure 1:**
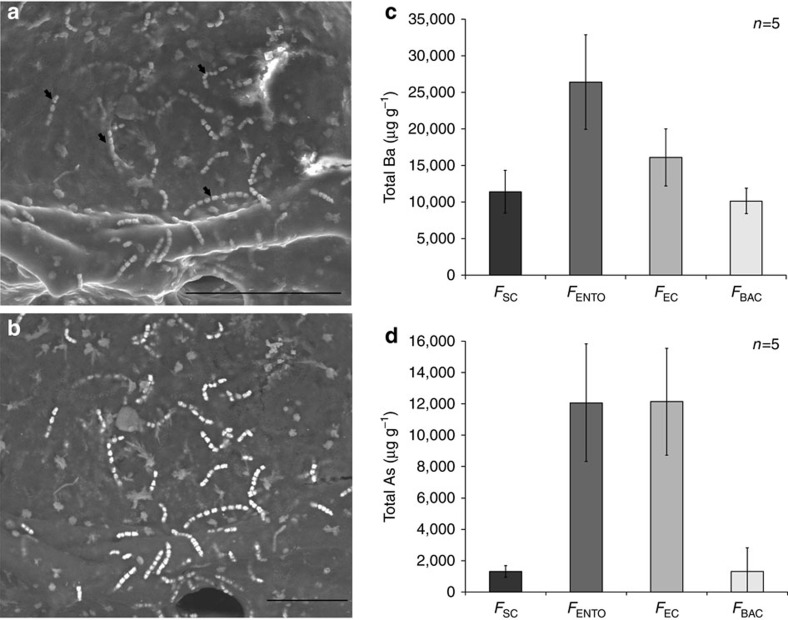
*Entotheonella* sp. is the arsenic and barium accumulating entity in the *T. swinhoei* holobiont. (**a**,**b**) Compatible SEM micrographs of *Entotheonella* sp. in sponge tissue. (**a**) Secondary electron detection mode with arrows indicating some of the *Entotheonella* sp. filaments. Scale bar, 100 μm. (**b**) Backscattered electrons detection mode reveals *Entotheonella* sp. filaments as the most-electron-dense objects in the sponge. Scale bar, 50 μm. (**c**,**d**) Dry weight concentration (μg g^−1^) of barium and arsenic (respectively) in cell-enriched fractions (*n*=5 samples, each one from a different sponge). *F*_BAC_, unicellular bacterial cells; *F*_EC_, *Entotheonella* sp. with cyanobacteria; *F*_ENTO_, *Entotheonella* sp. cells; *F*_SC_, sponge cells. Error bars show s.e.

Arsenic and barium have the highest atomic weight, among elements found in the sponge^14^. Thus, their accumulation can be detected using a scanning electron microscope (SEM) with backscatter detection mode (backscattered electron). Our preliminary results indicated that arsenic, and to some extent barium, were accumulated by the sponge-associated bacteria^18^. Surprisingly, when examining the sponge using SEM (*n*=4), we discovered that a single bacterium, identified as *Entotheonella* sp.^22^ (Fig. 1a), had the highest electron density (Fig. 1b). Using energy-dispersive X-ray spectrometry (EDS) we analysed their elemental composition and found that *Entotheonella* sp. contained both arsenic (6.07% weight ratio) and barium (11.7% weight ratio). The filamentous bacterium *Entotheonella* sp. was previously described in *T. swinhoei* as trichomes or chains^21^,^23^, with 4 to 20 cells. However, no analysis has been made to date of the electron-dense material observed in its cells, which from our analysis we now know to be rich in barium and arsenic. Next, we used inductively coupled plasma mass spectrometry (ICP-MS) to quantify arsenic and barium in four, easily separated, cell-enriched fractions: sponge cells (*F*_SC_), *Entotheonella* sp. (*F*_ENTO_) and unicellular bacteria (*F*_BAC_) from the inner layer. The fourth fraction was taken from the outer layer and contained *Entotheonella* sp. and cyanobacteria (*F*_EC_). The three fractions enriched from the inner layer (*F*_SC_, *F*_ENTO_ and *F*_BAC_) were tested against each other (Supplementary Table 1). As the two samples containing *Entotheonella* sp. (*F*_ENTO_ and *F*_EC_) were not independent, they were compared separately (Supplementary Table 2). We found the highest concentration of barium in *F*_ENTO_ (26,420±6,450 μg g^−1^ cells dry weight, *n*=5; Fig. 1c). Permutated analysis of variance (ANOVA) followed by Tukey's *post hoc* grouping showed *F*_ENTO_ barium concentration to be significantly higher than *F*_BAC_ (Supplementary Table 1). The difference between *F*_ENTO_ and *F*_SC_ was also tested by an exact permutation test, which showed they were significantly different (*P*=0.024). All cell fractions contained a high arsenic concentration compared to other marine taxa^3^, with fractions containing *Entotheonella* sp. (*F*_ENTO_ and *F*_EC_) displaying the highest concentration (*F*_ENTO_: 12,072±3,740 μg g^−1^ cells dry weight, *n*=5; *F*_EC_: 12,142±3,406 μg g^−1^ cells dry weight, *n*=5; Fig. 1d). Permuted ANOVA followed by Tukey's *post hoc* grouping showed arsenic concentration in *F*_ENTO_ was significantly higher than those of the other inner fractions (Supplementary Table 1). We thus deduced that *T. swinhoei* either actively retains arsenic^14^ or passively uptakes and sequesters at a greater rate than it can excrete^24^.

Our examination revealed that *Entotheonella* sp. comprises 3.25% (s.e.=0.0987) of the sponge volume (see calculation in Methods), and has a volume/weight ratio of 0.478. From this, we calculated that a 100 ml sponge would include 1.55 g of *Entotheonella* sp., containing 7,110 μg of water-soluble arsenic (Supplementary Table 2). This is equivalent to the amount of arsenic found in 2,370 l of seawater (according to the recorded concentration of 3 μg l^−1^; ref. 18). At maximum pumping rate^25^, such a specimen can filter 288 l of seawater daily, reaching a maximum arsenic exposure of 864 μg. Thus, we propose that *Entotheonella* sp. acts as the accumulating and detoxifying entity in the sponge holobiont.

### *Entotheonella* sp. mineralizes barium and arsenic

Biomineralization is usually described as either biologically controlled mineralization (BCM) or biologically induced mineralization (BIM), although intermediate forms are known^26^,^27^,^28^. BIM generally occurs extracellularly when metabolic byproducts react with chemicals in the environment. Minerals formed this way have poor crystallinity and are often unspecific. Intracellular BIM can occur in some bacteria, even up to the point of cell lysis^29^,^30^, but this occurs mostly in environments enriched with metal ions. These ions are mainly associated with sulfide, and precipitation is considered to be a detoxification mechanism^28^. BCM also exist in bacteria, such as magnetotactic bacteria, though is far less common than BIM. In BCM, minerals are deposited intracellularly on or within organic matrices or vesicles. The intravesicular conditions are controlled by the bacteria and are not affected by the environment. The minerals formed by BCM are well-ordered and specific.

**Figure 2:**
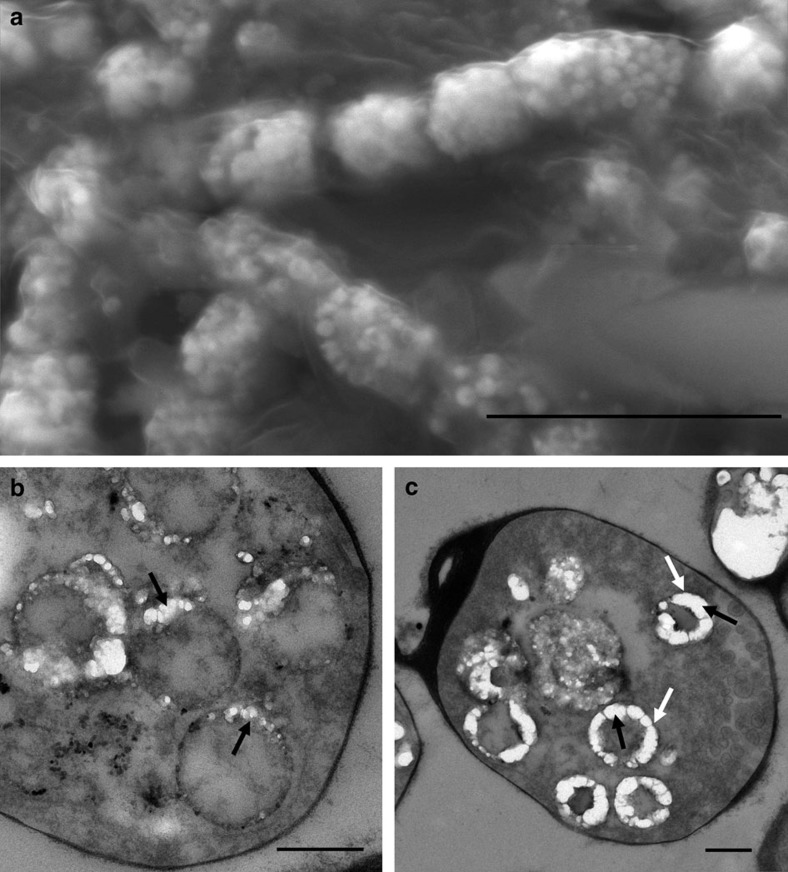
Intracellular mineralization in *Entotheonella* sp. (**a**) SEM micrograph (secondary electron mode) of *Entotheonella* sp. showing mineralized spherical structures inside their cells. Scale bar, 10 μm. (**b**,**c**) TEM micrographs of thin sections of *Entotheonella* sp. Black arrows in **b** mark nucleation of minerals from the vesicles' membrane. Black arrows in **c** mark the thickening of the mineral inwards from the membrane of the vesicle (white arrows). Scale bar, 500 nm.

A close look at *Entotheonella* sp. filaments from *T. swinhoei* shows that they contain multiple spherical granules inside their cells (Fig. 2a). The sphere volume is quite homogenous, averaging 0.11±0.007 μm^3^ with a median volume of 0.08 μm^3^ (*n*=121; Supplementary Fig. 1). Observation of thin sections of *Entotheonella* sp. under transmission electron microscopy (TEM) revealed that mineralization nucleates from the membrane of vesicles (Fig. 2b), thickening inwards (Fig. 2c).

**Figure 3:**
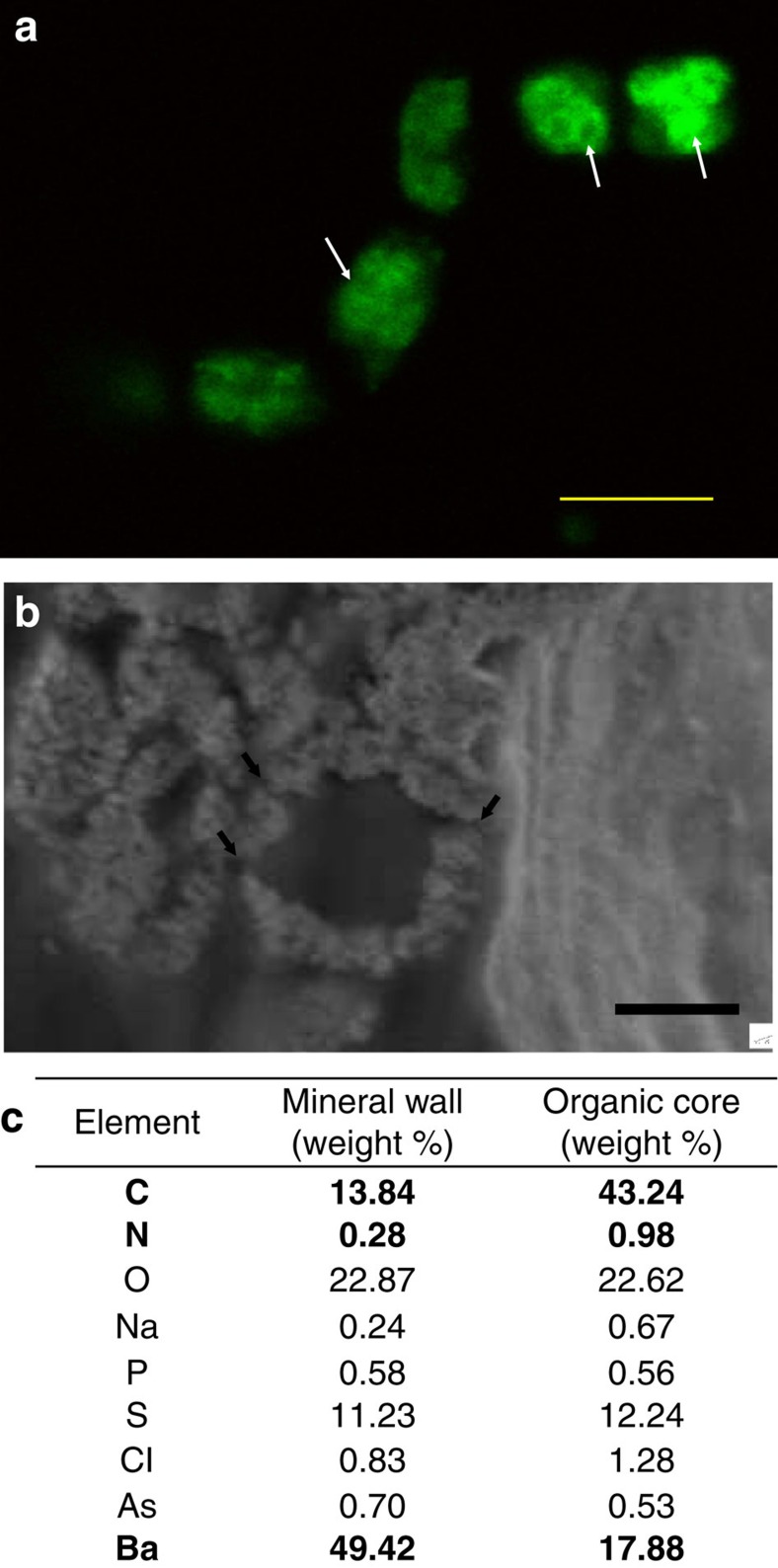
Characterization of spheres in *Entotheonella* sp. (**a**) Membrane staining with DiOC_6_ dye (excitation (ex), 482 nm; emission (em), 504 nm) shows that the vesicles (marked by arrows) of *Entotheonella* sp. are lipid based. Scale bar, 5 μm. (**b**) SEM micrograph (secondary electron) of cross-section of sphere in *Entotheonella* sp. The sphere wall is highly porous, with resemblance of channels (marked by arrows). Scale bar, 200 nm. (**c**) Average weight ratios (*n*=3) of detected elements determined by EDS. Carbon (C), nitrogen (N) and barium (Ba) are marked in bold.

To verify that the vesicle membranes are lipid-based, live *Entotheonella* sp. cells (*n*=5) were stained with the fluorescent membrane stain, 3,3′-dihexyloxacarbocyanine iodide (DiOC_6_) (Fig. 3a). Focused ion beam (FIB) cross-sections of the bacterium (*n*=1) were prepared for SEM-EDS analysis. High-resolution imaging of the cross-sections shows that the mineral ‘wall' is highly porous (Fig. 3b). We hypothesize that the porous nature of the sphere wall may facilitate material exchange between the core of the vesicle and the cytoplasm, via the membrane. Analysis of the elemental composition of the mineral wall and the vesicle core was conducted (Fig. 3c). Barium was significantly higher in sphere wall than in the core (two-sample permutation test using Welsh's *t*-test, two-sided test, *n*=3, *t*=−3.6213, *P* value <2.2e−16). Arsenic however was similar between wall and core. The core contained more organic matter, as indicated by a substantially higher carbon presence (two-sample permutation test using Welsh's *t*-test, one-sided test, *n*=3, *t*=4.4772, *P* value=0.0471). Nitrogen content in the core was also significantly elevated (two-sample permutation test using Welsh's *t*-test, two-sided test, *n*=3, *t*=3.8697, *P* value=0.0474), which might indicate the potential presence of proteins.

To determine the biomineral phase composition in *Entotheonella* sp., we analysed spheres using high-resolution synchrotron X-ray powder diffraction (XRD) in freeze-dried samples (*n*=5). We found that the crystalline fraction contains several minerals.

**Figure 4:**
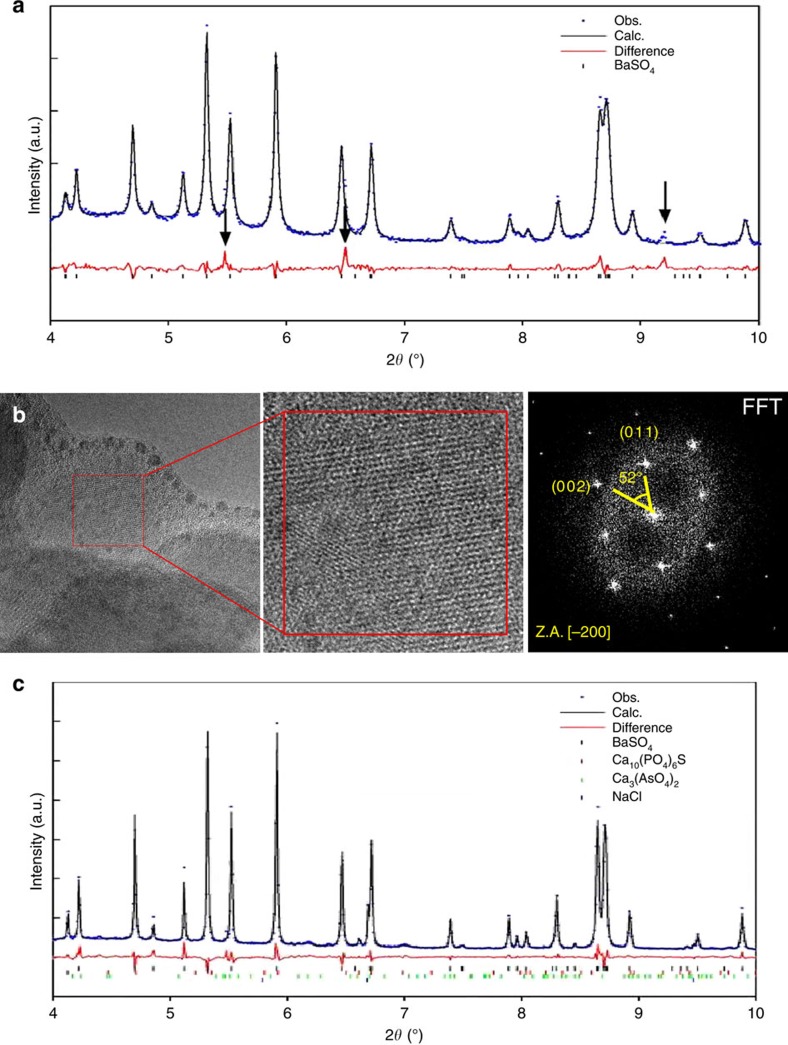
XRD and TEM analysis of spheres inside *Entotheonella* sp. (**a**) Rietveld refinement plot of preheated freeze-dried *Entotheonella* sp. reveals the resulted fit for the observed (blue dots) and calculated (black line) diffraction patterns and the difference between them (red line). Black notches indicate the positions of the diffraction peaks of crystalline barite (BaSO_4_), the major phase in the sample. Diffraction peaks highlighted by arrows in the difference curve are compatible with an arsenate or phosphate phase. These minor phases are not clearly visible in the observed diffraction pattern as they overlap with the diffraction peaks of barite. (**b**) HRTEM micrograph of a portion of the freeze-dried *Entotheonella* sp. Insert shows higher magnification image revealing the lattice, and with its Fast Fourier Transform (FFT). (**c**) Rietveld refinement plot of a freeze-dried *Entotheonella* sp. after thermal annealing, revealing minor phases of sodium chloride (NaCl), calcium arsenate (Ca_3_(AsO_4_)_2_) and calcium sulfide phosphate (Ca_10_(PO_4_)_6_S). Observed (Obs.), calculated (Calc.) and difference curves are presented in the same schematics as in **a**.

Barite was the major phase, with arsenates and phosphates probably minor phases (Fig. 4a). Crystalline barite presence within spheres was also confirmed by high-resolution TEM examination of FIB-prepared thin section (Fig. 4b). The fast Fourier transform of the TEM image obtained from a magnification close to an individual atomic lattice layer exhibits a single crystal electron diffraction from [−2 0 0] zone axis of barite. *Entotheonella* sp. is thus the only known prokaryote capable of intracellular barite mineralization.

The detected barite was polycrystalline, and a precise structural determination of the minor phases could not be achieved due to their poor crystallinity and the high organic matter content present in the freeze-dried samples. Aiming to improve the quality of the crystals and to eliminate the organic phase, we further subjected the samples to mild thermal annealing (250 °C for 2 h). The diffractogram collected after heating allowed us to detect crystalline calcium arsenate (Ca_3_(AsO_4_)_2_) and calcium sulfide phosphate (Ca_10_(PO_4_)_6_S) as minor phases (Fig. 4c). Quantitative phase analysis performed using the Rietveld method^31^ enabled the precise quantification of each crystalline phase (weight fractions) comprising the investigated samples: BaSO_4_ accounted for 87% of the crystalline weight, Ca_3_(AsO_4_)_2_ for 7% and Ca_10_(PO_4_)_6_S for 6%.

**Figure 5:**
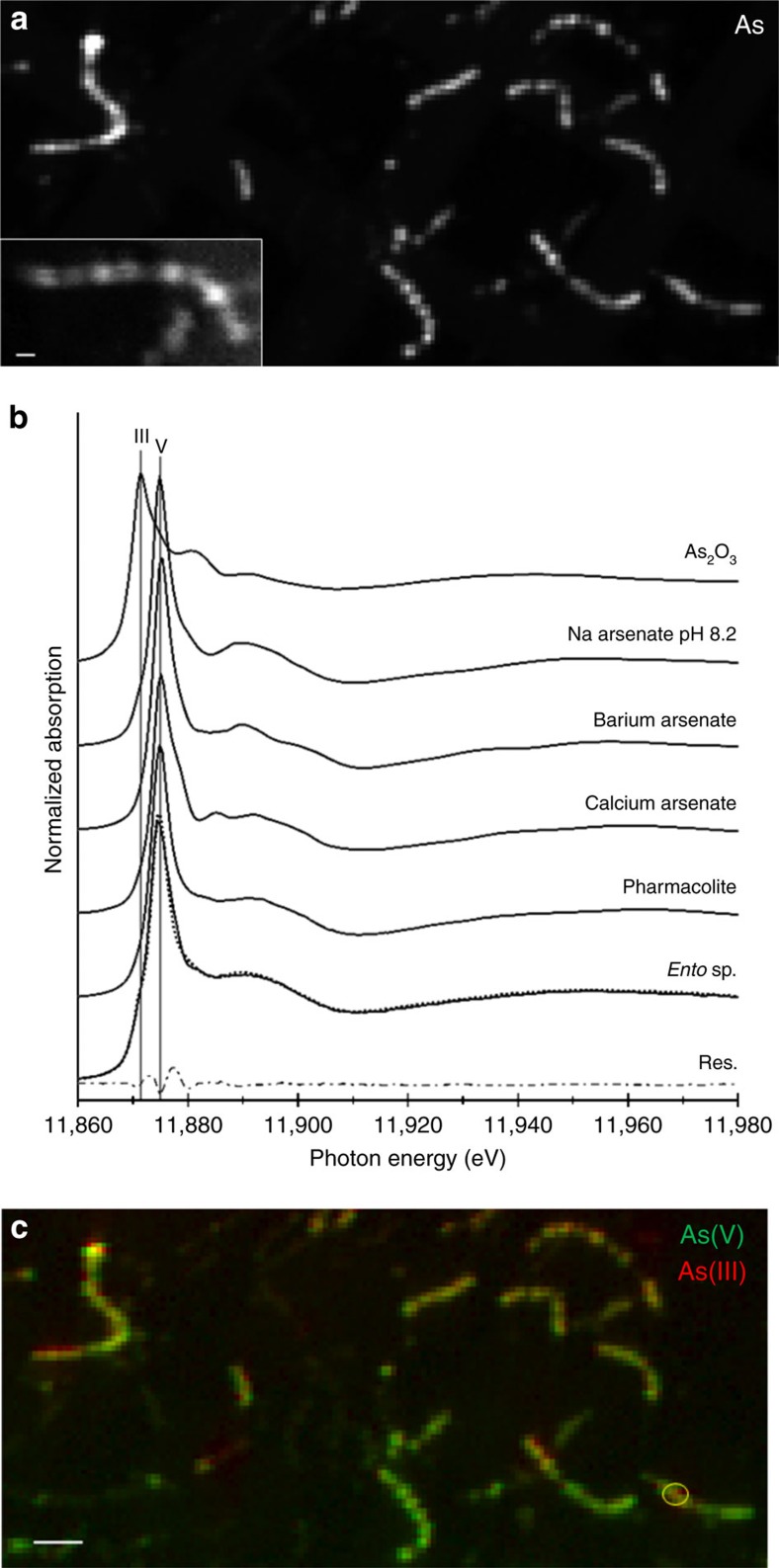
X-ray microprobe analysis of arsenic in *Entotheonella* sp. at 95 °K. (**a**) X-ray fluorescence distribution map of As in filaments. Pixel size is 2 μm. Inset: As map of a single filament, pixel size is 0.8 μm, scale bar, 4 μm. (**b**) As K edge spectrum of *Entotheonella* sp. compared with As(V) and As(III) standards. Least-square linear combination fitting using 47% sodium arsenate, 39% pharmacolite and 14% As_2_O_3_ is shown as dotted line (residuals in dashed line, norm. sum-sq=7.6e^−4^). (**c**) X-ray fluorescence chemical map showing the distribution of As(III) and As(V) in filaments in **a** area. Yellow circle points to location of XANES analysis (**b**). Scale bar, 20 μm.

**Figure 6:**
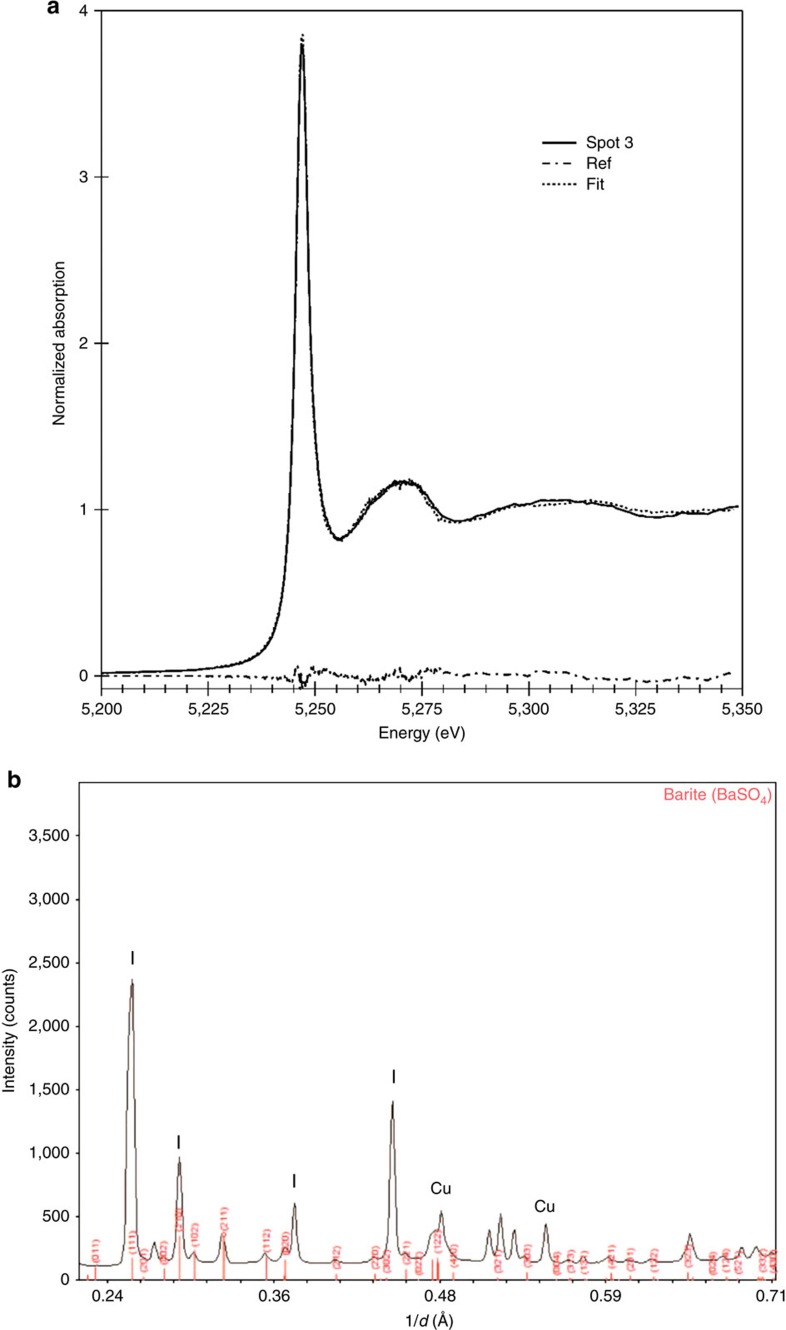
X-ray microprobe analysis of barium in *Entotheonella* sp. at 95 °K. (**a**) Ba L_3_ edge XANES on a filament (spot3) along with LCF fit (dotted line) to 100% barite (residuals in dashed line, norm. sum-sq=3.54e^−4^). (**b**) Indexing of the XRD pattern recorded at 17 keV at that location confirms the presence of crystalline barite. I- peaks from Ice crystals; Cu- peaks from copper grid.

The presence of barite and calcium arsenate were further confirmed on unprocessed ‘intact' flash-frozen *Entotheonella* sp. samples by synchrotron X-ray microprobe analyses (X-ray fluorescence mapping, X-ray absorption spectroscopy and XRD) performed at 95 °K. The distribution of arsenic in the filaments was evident by micro-focused X-ray fluorescence (μXRF) mapping (Fig. 5a and inset). All arsenic K edge X-ray absorption near edge structure (XANES) spectra of *Entotheonella* sp. showed As(V) as the dominant valence when compared to relevant standards (Fig. 5b). Generally, the white line increases in height, shifts towards higher energy and decreases in full-width at half-maximum as the oxidation state increases^32^. Least-square linear combination fitting (LCF) of 39 filament spectra (Fig. 5b) to a large library of 64 arsenic reference compounds from the ALS BL 10.3.2 database was performed. Top best LCF fits using 1, 2 or 3 components consistently required non-sulfur As(III), calcium arsenates (mostly pharmacolite) and sodium arsenate and best fits were obtained using combinations of these three components. μXRD performed on some of the filaments' XANES locations showed the presence of pharmacolite (Supplementary Fig. 3). The three-component LCF performed on 39 filaments indicated an average (rounded to full digits) of 18% As(III), 32% pharmacolite and 50% sodium arsenate. However, a precise determination of compound proportion could not be achieved for several reasons: the identity of the non-sulfur As(III) compound in unknown, the spectral fine structure of sodium arsenate varies with pH^33^ and concentration^34^ and fine spectral features vary with the degree of crystallinity. Compound abundance depended on location, as further evidenced by As(III)/As(V) chemical mapping, which exhibited heterogeneous distribution (Fig. 5c). LCF of Ba L_3_ edge XANES data on the filaments (Fig. 6a) best matched barite^35^ whose presence was further confirmed by μXRD (Fig. 6b). These combined results provide strong evidence of intracellular mineralization of barium (in the form of barite) and intracellular crystalline arsenic.

To determine whether the crystalline phases of barium and arsenic could explain all the measured elemental concentrations, we compared the atomic ratios of barium, sulfur and arsenic. Water-soluble element concentration (barium: 541 μg g^−1^ cells; sulfur: 1,084 μg g^−1^ cells; arsenic: 4,577 μg g^−1^ cells) was subtracted from the total elemental concentration and normalized by atomic weight. For crystalline barite (BaSO_4_) to explain all insoluble barium the Ba:S atomic ratio should be ≤1. While our measurements showed the ratio to be >1 (average=2.14±0.84, *n*=5), this is not significantly different from a ratio of 1 (paired *T*-test, *t*=1.4212, d.f.=4, *P* value=0.2283). Our analysis thus showed that the majority of insoluble barium can be explained by crystalline barite. This was further confirmed by Ba XANES and μXRD data (Fig. 6). Examination of crystalline arsenic is slightly more complicated. To do so, we calculated the weight ratio of crystalline barium and arsenic. Barium comprises 58.8% of barite molecular weight, and thus constitutes 51.2% of total crystalline weight. Arsenic comprises 37.7% of Ca_3_(AsO_4_)_2_ molecular weight, and thus constitutes 2.6% of total crystalline weight. From the Ba:As ratio (20:1), we calculated that 1,290 μg g^−1^ (or 17.2%) of the insoluble arsenic is crystalline. The poor crystallinity of arsenic and spatial variation of calcium arsenate and As(III) in the XANES fit prevents a clear identification of the amorphous arsenic solid. However, since the average ratio of calcium arsenate by XANES analysis is 32% (∼3,850 μg g^−1^ cells), we can say with confidence that some of the calcium arsenate is amorphous. The identity of the As(III) compound is unknown and may either be soluble or solid.

Altogether, the results indicate that *Entotheonella* sp. controls mineral formation within its cells to a relatively high degree. This phenomenon exhibits many of the characteristics of BCM, with the exception of the ability to mineralize finely ordered crystalline material. Thus, we conclude that *Entotheonella* sp. possesses an intermediate form of BCM, with some similarities to magnetotactic-like bacteria^28^.

### Bioavailability of arsenic in *Entotheonella* sp

We tested the presence of arsenate (as sodium arsenate) and a compound with As(III) using the XANES analysis (Fig. 5). Since most soluble arsenic species are extremely toxic, we identified and quantified them in the enriched cell fractions from the sponge (Table 1). We separated arsenic species using ion exchange chromatography and identified them against known standards (arsenite: As(III); arsenate: As(V); monomethylarsonic acid (MMA), dimethylarsinic acid (DMA), arsenobetaine (AsB) and arsenocholine (AsC)). As(V) and MMA were detected in all fractions. DMA and AsB were detected in *F*_SC_, *F*_EC_ and *F*_BAC_. As(III) was only detected in *F*_EC_. The absence of soluble As(III) in *Entotheonella* sp. means that the As(III) detected by XANES is either an amorphous solid or bound to the vesicle membranes, making it insoluble in water.

The dominant arsenic species within each fraction was tested by a permuted ANOVA, followed by a *post hoc* Tukey's test. We found that the arsenate concentration, as hypothesized previously^19^, was significantly higher than all other arsenic species in all cell fractions, except in *F*_BAC_ (*F*_**SC**_: 892.4±284.4 μg g^−1^ cells, *F*=9.797, *P* value=3.37e^−5^, *F*_ENTO_: 4,498±983.4 μg g^−1^ cells, *F*=20.7, *P* value=5.2e^−8^; *F*_EC_− 4,301±1,228 μg g^−1^ cells, *F*=12.06, *P* value=6.5e^−6^; *F*_BAC_: 747±485.7 μg g^−1^ cells, *F*=2.48, *P* value=0.0719).

Upon testing differences in arsenate concentration among fractions, we found that *F*_ENTO_ had a significantly higher arsenate concentration than all other cell fractions (permuted ANOVA, followed by *post hoc* Tukey's test, *F*=10.55, *P* value=0.00227). Bacteria are reported to tolerate as much as 1 M of arsenate (or 75,000 μg g^−1^ arsenic)^36^ by means of continuous excretion and maintenance of low intracellular concentrations^5^. Our analysis indicates that *Entotheonella* sp. accumulates, rather than excretes, arsenate. Moreover, a statistical test (exact permutation test estimated by 999 Monte Carlo replications) showed that arsenate concentrations in *Entotheonella* sp. (normalized by atomic mass) are significantly higher than those of water-soluble sulfur (912±205 μg g^−1^ cells, *P* value=0.024, confidence interval (CI)=0.00877, 0.04534) and phosphorous (641±169 μg g^−1^ cells, *P* value=0.01, CI=0.00135, 0.02511), both of which are essential elements^24^. Even with such high arsenate concentrations, *Entotheonella* sp. remains viable, as indicated by 5(6)-carboxyfluorescein diacetate *N*-succinimidyl ester (CFDA/SE) vital staining (Supplementary Fig. 2). If arsenic is essential to the metabolism^37^ of *Entotheonella* sp., we can reasonably expect to detect high arsenate concentrations within the cell. If *Entotheonella* sp. does not utilize arsenic metabolically, it should then possess highly efficient detoxification mechanisms. Mineralization of arsenic is one such mechanism, but with 4,577 μg g^−1^ water-soluble arsenate, other mechanisms must also exist. We hypothesize that the soluble arsenic is localized within the core of the vesicles since arsenate does not pass through lipid membranes^5^.

### Potential pathway for element accumulation in *Entotheonella*

**Figure 7:**
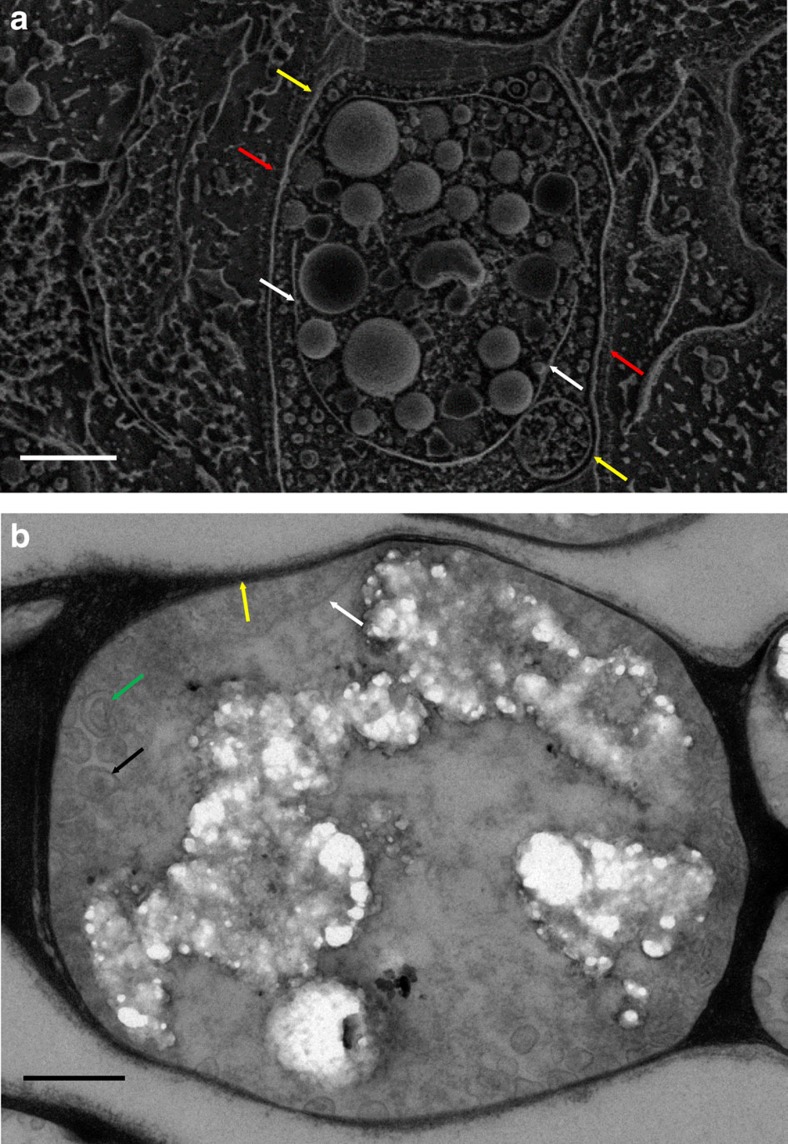
Membrane system of *Entotheonella* sp. (**a**) Cryo-SEM micrograph of freeze-fractured *Entotheonella* sp. filament. Red arrows mark the filament sheath. Yellow arrows mark the outer membrane. White arrows mark the internal membrane harbouring the mineralized spheres. Scale bar, 1 μm. (**b**) TEM micrograph of thin sections of *Entotheonella* sp. showing vesicles in the space between the two membranes. Yellow arrow marks the outer membrane. White arrow marks the internal membrane. Black arrow marks a single membrane vesicle. Green arrow marks a double membrane vesicle. Scale bar, 500 nm.

To achieve mineralization, *Entotheonella* sp. needs to transport and concentrate the relevant elements at the site of mineralization^38^. *Entotheonella* sp. possesses an outer sheath, which surrounds the entire filament, and two sets of membranes (Fig. 7a). The mineralized vesicles are only found in the inner space. In the marine environment, both arsenate^3^ and barium ions^6^ are charged, so passive transport across an outer wall and two layers of lipid membranes is an unlikely explanation for the accumulation^39^. In bacteria, arsenate may enter cells via phosphate transporters^5^, and barium ions via K^+^ and Ca^2+^ channels^40^. Such unspecific transport mechanisms are also an unlikely accumulation path, since the aforementioned transporters favour their intended molecule. *Entotheonella* sp. may have yet undiscovered specific transporters for these ions. A third potential mechanism for ion transport and accumulation is pinocytosis of seawater^41^. Such a process is rare in bacteria, but there is some evidence of an endocytosis-like process in the genus *Gemmata*^42^.

Observation of *Entotheonella* sp. reveals that the space between the two membranes contains many small vesicles (Fig. 7b). Interestingly, some vesicles contain a single membrane while others have a double membrane (Fig. 7b, green arrow). We hypothesize that these vesicles may be involved in the transport of arsenic and barium between the immediate surroundings and the inner volume of *Entotheonella* sp. Arsenic may be linked to the membranes themselves as As(III), while barium ions would likely be in the bulk liquid.

Another noteworthy aspect of the accumulation process is the merging of discrete mineralized spheres (Supplementary Fig. 4a). These spheres are discrete in the majority of cell units in *Entotheonella* sp. filaments, but the merging of some spheres was also observed, in some cases affecting all mineralized material (*n*=9). Further, all cells in which full sphere merging occurred, seemed to have lost their inner membrane (Supplementary Fig. 4b).

## Discussion

Here we describe a host–bacterium relationship established around the accumulation and detoxification of harmful trace elements. Previously, we concluded that arsenic and barium were coaccumulated, based on statistical analyses that showed grouping of the aforementioned elements^14^. Here we demonstrate that both elements are localized to a specific symbiotic bacterium, *Entotheonella* sp. The combined results of previous^14^ and present research provides evidence supporting a linkage between arsenic and barium cycles in *T. swinhoei*, driven largely by *Entotheonella* sp. In past measurements of arsenic in Japanese sponges, a *Theonella* sp. was found to have the highest arsenic concentration among analysed species, with 157 μg g^−1^ (only water-soluble arsenic was measured)^43^. While this is much lower than the concentration we found in Red Sea *T. swinhoei*, it is still relatively high for sponges. Interestingly, the concentration of AsB in the Japanese *Theonella* sp. equals the concentration of AsB in *F*_EC_. It is compelling to speculate that at some point in the *T. swinhoei* evolution and dispersion towards the Red Sea, *Entotheonella* sp. evolved in a way that significantly affected the arsenic cycle of the sponge.

Multicellular organisms can detoxify arsenic by accumulating and precipitating the element in designated excretion and detoxification tissues^7^. *T. swinhoei* does not possess differentiated tissues but our results suggest an alternative detoxification mechanism. *Entotheonella* sp., residing within the sponge's mesohyl, can accumulate ten times more water-soluble arsenic than the sponge's daily exposure to soluble arsenic, which it then mineralizes. Therefore, we propose that *Entotheonella* sp. may function, to some extent, as a detoxifying organ for *T. swinhoei*. Excretion of *Entotheonella* sp. by the sponge is unlikely, as past measurements have shown very few bacterial cells in this sponge's excurrent^25^.

Other microorganisms reported to accumulate arsenic have a much lower arsenic concentration^44^ or maintain a high concentration due to elevated arsenic in the environment^45^. Total arsenic concentration in *Entotheonella* sp. (12,000 μg g^−1^ cells) is among the highest measured in any known organism^3^ or surficial deposit^46^. Furthermore, we show that *Entotheonella* sp. can form intracellular crystalline arsenic, and mineralize barium intracellularly. The only other arsenic biogenic mineral so far described is orpiment^38^, thus biomineralization of calcium arsenate by *Entotheonella* sp. adds to the ever growing list of biominerals. Another prokaryote (*Desulfotomaculum auripigmentum*) precipitates arsenic intracellularly^47^, but amorphously, localized near the cell membrane and eventually excreted. Our analyses indicate that *Entotheonella* sp. mineralizes arsenic and barium solely intracellularly, without excretion.

Still, some questions remain unanswered. The origin of the membranes on which the biomineralization occurs remains to be elucidated. Whether they result from invagination of the plasma membrane or constitute a separate synthesized membrane^48^ is unknown. The exact mechanism of element accumulation and transport to the site of mineralization is another subject for which future research may provide answers. However, it is clear that *Entotheonella* sp. possesses a unique bacterial system for element accumulation and mineralization. The formation of the biominerals might contribute to detoxification, but they could also play other, yet unknown functions.

## Methods

### Sample collection

We collected samples of *T. swinhoei* (*n*=12) from the Red Sea (34°55′02′′/29°60′05′′), by SCUBA diving at 15–30 m depth (permit no.: 2013/40172; Israel Nature and Parks Authority). Sponges were identified by visual and tactile characteristics underwater. In the lab we further inspected the inner tissue visually. Final identification was based on the presence of *Entotheonella* sp. and typical microdesma spicules under light microscope. We processed the samples immediately after collection at the Interuniversity Institute for Marine Sciences in Eilat, Israel. Work was performed under sterile conditions in a laminar flow hood. Before processing we thoroughly rinsed the sponges with sterile calcium-magnesium-free artificial seawater (CMF-ASW-NaCl 26.22 g, KCl 0.67 g, Na_2_SO_4_ 4.62 g, NaHCO_3_ 0.21 g, Na EDTA 0.37 g, ddH_2_O 1 L set to pH 8) to wash off epibionts and transient bacteria.

Methods for cell separation and subsequent quantitative analysis, XRD, staining, X-ray microprobe analysis and cryogenic scanning electron microscopy (cryo-SEM) are described below.

### SEM-EDS sample preparation and analysis

We cut small pieces of sponge samples for energy dispersive X-ray spectrometry with SEM (SEM-EDS) using sterile scalpel blades. We included the cyanobacteria-rich outer layer and the dense endoderm. We fixed the samples in 2.5% glutaraldehyde (in CMF-ASW) and kept them in the dark at 4 °C until used. Fixed sponge samples were dehydrated by an ethanol gradient series (50% EtOH for 30 min, 70% EtOH 8 h, 100% EtOH 30 min), followed by critical point drying and coating with carbon. Imaging and elemental analysis were performed using a High-Resolution SEM (HR-SEM; ULTRA Plus; Zeiss, Oberkochen, Germany) equipped with INCA (Oxford Instruments, England) EDS. SEM images were captured at different acceleration voltages using an Evehart Thornley secondary electron detector, enabling investigation of the surface topography and an energy selective backscatter detector suiTable for obtaining a clear compositional contrast (Z-contrast). EDS spectra were collected at points of interest using the accelerating voltage of 10 keV at the working distance of 6 mm. Quantitative analysis was performed using the conventional correction procedure included in the INCA software. Final results were normalized to 100% and presented as a relative ratio of elements mass (weight %). Cross-sectioning of *F*_ENTO_ was performed using an FEI Strata 400S dual-beam FIB, which is a fully digital field emission scanning electron microscope equipped with FIB technology. FIB effectively enables the preparation of cross-sectioned samples by cutting a particular part of the examined object in the required direction.

### Cell separation

We separated *T. swinhoei* (*n*=5) into enriched cellular fractions based on well-established differential centrifugation protocols^22^,^49^, with some changes. First, we cut off the outer part, containing cyanobacteria, from the inner part of the sponge, using a sterile scalpel (further processing of the two parts was done separately).

We divided the inner part of the sponge into three cell-enriched fractions: sponge-enriched fraction (*F*_SC_), *Entotheonella* sp.-enriched fraction (*F*_ENTO_) and unicellular bacteria-enriched fraction (*F*_BAC_). From the outer part of the sponge, we separated a fourth fraction, containing *Entotheonella* sp. and cyanobacteria (*F*_EC_). We used all four cell fractions, from five sponge samples, for quantitative elemental analysis. From the same sponge samples, we took aliquots of *F*_ENTO_ for XRD, TEM and cell staining. We collected the *Entotheonella* sp. fraction for cryo-SEM from two independent sponge samples. *Entotheonella* sp. for X-ray microprobe analysis originated from two additional independent sponge samples.

To enrich each fraction, we homogenized sponge samples in a juicer (Moulinex, France) with 1 l of CMF-ASW and agitated the homogenate by stirring to disassociate cells. Cell agitation was conducted over three rounds of 2 min stirring and 2 min rest, ending with 10 min settling time. After settling, we filtered the supernatant through a nylon mesh (50 μm pore size) into a clean collection conical flask. We resuspended the settled material in 1 l of CMF-ASW and repeated the agitation and filtering steps, adding the filtered supernatant from the second round to the collection conical flask. The leftover settled material, after the two rounds of agitation and filtering, was the fraction enriched in sponge cells (fraction *F*_SC_). We continued to separate the bacterial fractions from the supernatant. From the inner parts of *T. swinhoei*, we pelleted fraction *F*_ENTO_ by centrifugation of the supernatant at 150*g*, and transferred the supernatant to a new tube. We pelleted fraction *F*_BAC_ by centrifugation of the supernatant at 7,000*g*, discarding the supernatant at the end. From the outer parts of *T. swinhoei* we pelleted fraction *F*_EC_ by centrifugation of the supernatant at 300*g*, discarding the supernatant at the end. All cell fraction samples for quantitative and XRD analysis were kept at −80 °C, followed by lyophilization. Samples for staining, cryo-SEM and X-ray microprobe analysis were kept in CMF-ASW in the dark at 4 °C until used (within 24 h of sampling). Samples for TEM were fixed in 2.5% glutaraldehyde (in CMF-ASW) and kept in the dark at 4 °C until used.

### Computation of dry weight to volume for *Entotheonella* sp

We calculated the volume of *Entotheonella* sp. in the sponge from image analysis of histological slides (six slides prepared in previous years^20^) of two sponge specimens. We obtained at least 11 images at magnification × 20 from randomly chosen areas of the sponge section of each slide (total of 67 images). The total sponge volume was calculated from the area observed and a depth of 2 μm (the diameter of *Entotheonella* sp. filaments). The surface area of *Entotheonella* sp. in a given image was measured using the ICY software^50^ and the volume, assuming a cylinder shape. To compute the weight-to-volume ratio of *Entotheonella* sp., we collected a 0.25 ml bacterial pellet by centrifugation and measured its weight after lyophilization.

### Quantitative elements analysis

Form each cell fraction, we aliquoted a 600 mg dry weight sample and sent it to Brooks Applied Labs (WA, USA) for elemental analysis. The following measurements were conducted: total elemental concentration of arsenic, barium, sulfur and phosphorous; total water-soluble elemental concentration of arsenic, barium, sulfur and phosphorous; speciation of water-soluble arsenic forms.

### Total element extraction for analysis

Elements were extracted by nitric acid (HNO_3_) digestion, following the EPA method 3050B^51^ with 50–100 mg (dry weight) of the cell fraction. Samples were placed in polypropylene centrifuge tubes and heated in concentrated HNO_3_ to 95 °C for 15 min on a hot-block apparatus. Once cooled, additional HNO_3_ was added and the samples were reheated to 95 °C for 30 min, during which the formation of brown fumes was observed. This step was repeated until no fume formation was detected, and ended with 2 h incubation at 95 °C. Once acidic digestion was completed, H_2_O_2_ (30%) was added to the solution in aliquots and gassing was monitored until it subsided to a minimum, or sample appearance remained unchanged. An additional 2 h of incubation was allowed to reduce solution volume before it was cooled to room temperature. Before analysis, samples were diluted in ddH_2_O and filtered to remove any particulate matter.

### Water-soluble element extraction for analysis

Extraction was based on a previously published protocol^43^ and conducted with 50–100 mg (dry weight) of the cell fraction. Samples were placed in polypropylene centrifuge tubes and soluble compounds were extracted in 10 ml of water:MeOH (1:1 v v^−1^) by shaking at 80 r.p.m. for 2 h. The sample was then centrifuged and divided in half. Half of the supernatant (5 ml) was filtered (0.45 μm) and directly measured. The other half was transferred with the pellet to a hot-block apparatus (set at 95 °C) for 3 h, followed by centrifugation and filtration of the supernatant (0.45 μm) for measuring. The results obtained from the two water-methanol extracts associated with each sample were combined to reflect the overall extractable fraction of a given analyte.

### Separation and detection of arsenic species for analysis

Aliquots of each water-methanol extract were injected onto an anion exchange column and mobilized by an alkaline (pH>7) gradient, following a described protocol^52^. Retention time for each detected arsenic species in the samples was compared to those of known standards for species identification: (1) arsenate (AsV); (2) arsenite (AsIII); (3) monomethylarsonic acid (MMA); (4) dimethylarsinic acid (DMA); (5) AsB; (6) arsenocholine (AsC). All other arsenic species identified were defined as unknown arsenic species.

Three sets of laboratory control samples and matrix spikes were prepared during the speciation extraction to monitor any potential species conversion attributable to either the applied extraction procedure or the sample matrices. The first set contained AsIII, MMA, DMA and AsB; the second set contained only AsV; and the third set contained only AsC. In addition to arsenic species, phosphate and sulfate from *F*_ENTO_ were separated and quantified as well, following the protocols described above.

### Quantitative analysis of elements and element species

Quantification concentration was done using ICP-MS. All samples analyses were preceded by a minimum of a five-point calibration curve spanning the entire concentration range of interest. The calibration curves associated with each element were standardized by linear regression, resulting in a response factor. All results were instrument blank corrected to account for any operational bias. Ongoing instrument performance was monitored by the analysis of continuing calibration verification standards and continuing calibration blanks. Results are presented as average with s.e.

### Analysis of *Entotheonella* sp. by TEM

To analyse cell structure with TEM, *Entotheonella* sp. fixed in 2.5% glutaraldehyde (in CMF-ASW) were washed twice in 50 mM phosphate buffered saline (PBS) (pH=8.0) and the cells were postfixed in 1% OsO_4_ in PBS for 2 h at 4 °C. Dehydration was carried out in graded ethanol followed by incubation in propylene oxide and embedding in glycidyl ether at the Instrumentation and Service Center of the Department of Life Sciences, Tel Aviv University. Thin sections were mounted on Formvar/Carbon-coated grids. Sections were stained with uranyl acetate and lead citrate and viewed under TEM (FEI Tecnai, USA) at the Weizmann Institute Electron Microscopy Unit.

Analysis of crystalline material using TEM was conducted on freeze-dried *Entotheonella* sp. A TEM sample of *F*_ENTO_ was prepared using an FEI Strata 400S FIB equipped with a high-precision piezoelectric specimen stage with a 100 mm stroke along the *x* and *y* axes, an *in situ* nano-manipulator (Omniprobe; AutoProb 200) sample extraction system for lift-out TEM specimen preparation, and flip-stage pivoting TEM grid mount. TEM investigation was carried out at the Technion, using a Titan FEI (S)TEM with a dedicated platform for corrector and monochromator technologies, which enables a resolution of 0.7 Å. Imaging was performed at the acceleration voltage of 300 keV.

### Membrane staining

We stained membranes of *Entotheonella* sp. filaments using DiOC_6_ following a protocol developed for other bacteria^53^. We incubated live cells in CMF-ASW with 2.5 μg ml^−1^ DiOC_6_ for 15 min (shaking in the dark at room temperature). Following incubation, we washed the cells and mounted them on glass slides in CMF-ASW. We observed the cells using a confocal microscope (Zeiss). Fluorescence of DiOC_6_ was detected at an excitation of 482 nm and emission of 504 nm. The fluorescence image was overlaid on a DIC image to better localize the stain in the filaments. Additionally, a Z-stack series (17 slices of 0.3 μm) with only fluorescent filters were acquired for selected filaments.

### XRPD of *Entotheonella* sp. and Rietveld analysis

An aliquot of *F*_ENTO_ was used for XRD analysis. Powders were characterized by high-resolution X-ray powder diffraction (XRPD) utilizing a synchrotron source. Diffraction measurements were conducted at the ID22 beam line of the European Synchrotron Research Facility (ESRF, Grenoble, France) equipped with a double-crystal monochromator and crystal analyser optical elements in the incident and diffracted beams, respectively, at a wavelength of 0.476798(8)  Å. Instrument calibration was performed using NIST silicon standards, and final instrumental contribution to the full-width at half-maximum did not exceed 0.004° 2*θ* (ref. 54). The samples were loaded into 1 mm diameter borosilicate glass capillaries rotating during the measurements at a rate of 60 r.p.s. to avoid intensity spikes from the individual grains subjected to quasiparallel beam irradiation. The diffracted beam was monochromatized and collected using a multianalyser stage equipped with a nine-point detector (for further information see, http://www.esrf.eu/id22/technical-description). Nine diffractograms, obtained from each detector, were binned in a 2*θ*/intensity diffractogram. The use of the advanced analysing optics resulted in diffraction spectra of superior quality.

Rietveld quantitative phase analysis was applied to collected diffractograms to determine weight quantity of the crystalline phases present in the *F*_ENTO_ sample. The analysis was performed using the GSAS-II software^55^.

### X-ray microprobe

*Entotheonella* sp. samples for hard X-ray microprobe analyses under cryogenic conditions were flash frozen onto carbon-coated copper 200-mesh grids at the EM unit (Weizmann Institute of Science, Israel)^56^. Grids were glow discharged for 45 s before application of sample. Cells in CMF-ASW were resuspended until reaching homogenous suspension and then 5 μl were placed on the grid. Grids were blotted (5 s) and plunged into liquid ethane using a Leica EM-GP automated plunger (Leica Microsystems) and stored in liquid nitrogen until measurements. Arsenic and barium bearing standard compounds were either mounted on carbon-coated copper 200-mesh grids TEM Cu grids or on Si_3_N_4_ windows (SiMPore Inc.). Three *Entotheonella* sp. sample grids with a minimum of two regions per grid were analysed.

μXRF mapping, μXRD and X-ray absorption spectroscopy (XAS) measurements were conducted at ALS bending magnet beamline 10.3.2 (2.1–17 keV) with the storage ring operating at 500 mA and 1.9 GeV (ref. 57). All data were recorded at 95 °K using a custom cryogenic setup (Instec Inc.) that allows for the transfer of frozen samples following procedures described in details elsewhere^58^. Radiation damage was not observed under these conditions. μXRF elemental distribution maps and μXRF spectra on each pixel were collected at 12,066 eV. Few elemental maps were also recorded at 5,347 eV above the Ba L_3_ edge to better detect Ca, P and S. Beam spot size was 3 × 3 μm^2^ with pixel size 1 × 1 μm^2^ or to 2 × 2 μm^2^ and counting times 80–120 ms per pixel. Fluorescence emission counts were recorded using a 7-element Ge solid-state detector (Canberra) and XIA electronics. Arsenic chemical maps were taken in multienergy per line mode, at 11,830 (pre-edge background), 11,868 (As(-I) sulfides) and 11,869 (As(III) sulfides), 11,871 (arsenite), 11,875 (arsenate) and 11,979 eV (postedge, total As). These energies were chosen to investigate the distribution of As(III), As(V) and sulfides, but do not allow to distinguish sodium arsenate from calcium arsenate. Chemical maps shown were deadtime corrected and fitted using As_2_O_3_ as a proxy for As(III) and pharmacolite as a proxy for As(V).

Arsenic K edge μXAS spectra were recorded in fluorescence mode by continuously scanning the Si (1 1 1) monochromator (Quick XAS mode) from 11,774 to 12,580 eV, with 0.5 eV steps in the XANES region. Spectra were calibrated using the white line of a Na_2_HAsO_4_ powder standard set at 11,875 eV, recorded at room temperature in transmission mode. All data were processed using the LabVIEW custom BL 10.3.2 software and standard procedures described elsewhere^59^. All reference and sample XANES spectra were carefully postedge normalized up to 12,178 eV. Least-squares linear combination fitting of the XANES spectra^60^ was performed using a large spectral database of arsenic compounds (64 compounds) from ALS beamline 10.3.2 and procedures described elsewhere^58^.

Arsenic standards we used: sodium arsenate at pH 8.2 (aqueous 1 M, Na_2_HAsO_4_·7H_2_O; Sigma; CAS no. 10048-95-0), calcium arsenate (powder, As_2_Ca_3_O_8_; Alpha Chemicals; CAS no. 7778-44-1), pharmacolite (crushed mineral, CaHAsO_4_·2(H_2_O), provided by the Mineral Collection of the Earth and Planetary Science Department, UC Berkeley) and barium arsenate (precipitate). Barium arsenate was prepared in the lab and made from a BaCl_2_ solution (Sigma; 0.1 M; CAS no. 10361-37-2) and sodium arsenate (Sigma). As(V) was dissolved in diH_2_O to a concentration of 0.1 M. Equal volumes of the solutions were mixed and precipitation followed. The precipitate was collected by centrifugation and freeze-dried. Barium and sodium arsenate were recorded at 95 °K in transmission mode; pharmacolite and calcium arsenate were recorded at 95 °K in fluorescence mode. As_2_O_3_ was recorded at room temperature in transmission mode.

Microdiffraction patterns were collected in transmission mode with a CCD camera (Bruker APEX2) at 17 keV (*λ*=0.729 Å) using a beam spot size of 12 × 6 μm^2^ and exposure time of 240 s. Calibration of the camera distance was obtained using an alumina (α-Al_2_O_3_) powder standard and Fit2D software^61^. Fit2D was also used to obtain one-dimensional XRD profiles from the radial integration of 2D patterns. These data were then indexed using Jade 9 software (Materials Data Inc.) and the ICDD PDF-4+ and MINCRYST crystallographic databases.

### Vitality staining of *Entotheonella* sp

We stained *Entotheonella* sp. filaments with CFDA/SE^62^ to assess their viability. We pelleted cells from CMF-ASW and resuspended them in PBS, adding CFDA/SE (50 mM in dimethylsulphoxide) to a final concentration of 100 μM. We followed this by stirring the suspended cells and cycling the temperature between 25 °C and 37 °C (15 min each) for 2.5 h. After temperature treatment we harvested the cells by centrifugation, washed and incubated them in marine broth (5 g l^−1^ bactopeptone, 1 g l^−1^ yeast extract in ASW, pH=8.2) at 15 °C with shaking for 48 h. Following incubation, we again harvested cells by centrifugation, washed them in PBS and mounted them on glass slides in 70% glycerol CMF-ASW. We observed the cells with a confocal microscope (Zeiss) for fluorescence at an excitation wavelength of 492 nm and emission of 517 nm. The fluorescence image was overlaid on a differential interference contrast (DIC) image to assist localization of the stain in the filaments.

### Cryo-SEM of *Entotheonella* sp

Sample preparation and observation was conducted at the Electron Microscopy Unit at the Weizmann Institute of Science. We transferred a live sample of *Entotheonella* sp. in CMF-ASW to the EM unit within 24 h of sampling. There we preserved the sample by high-pressure freezing (HPM 10; BAL-TEC, USA). Before observation, we fractured the samples in a freeze fracture device (BAF 60; BAL-TEC, USA). We observed *Entotheonella* sp. in an Ultra 55 fully digital field emission scanning electron microscope microscope (Zeiss) with a Leica cryo stage (AG, Liechtenstein, Germany), exposing the filaments by light heat milling.

### Statistical analysis

Sample size for quantitative analysis (total element concentration and arsenic species) was decided after a preliminary test with minimal sample size (*n*=3). Following the analysis, separation methods were further optimized so the original samples were not included analysis.

Statistical analysis was performed using R statistics^63^ with RStudio IDE (RStudio, USA). Significant values for all tests were regarded for *P* value <0.05. All measurements were tested for their fit to normal distribution (Shapiro–Wilk test). When we could not assume normal distribution, parametric tests with permutations were conducted. Permutations enabled the use of the stronger parametric tests using the true distribution of the sample by subsampling.

Differences in total arsenic concentration, total barium within arsenic species and among arsenate were tested by permuted ANOVA, using the lmPerm package^64^, and Tukey's *post hoc* test to assign groups of significance.

Difference in sphere volume, in total barium concentration between *F*_ENTO_ and *F*_SC_, differences in weight% (Ba, C, As and N) between the mineral wall and core, and difference in atomic or weight ratios of elements (Ba:S, Ba:As, As(V):S(aq), As(V):P(aq)) were tested using permutation test based on T distribution, using Perm and Deducer.

### Data availability

The authors declare that all relevant data supporting the findings of this study are available within the article and its Supplementary Information Files, or from the corresponding author on request.

## Additional information

**How to cite this article:** Keren, R. *et al*. Sponge-associated bacteria mineralize arsenic and barium on intracellular vesicles. *Nat. Commun.* **8,** 14393 doi: 10.1038/ncomms14393 (2017).

**Publisher's note:** Springer Nature remains neutral with regard to jurisdictional claims in published maps and institutional affiliations.

## Supplementary Material

Supplementary Information Supplementary Tables and Supplementary Figures

## Figures and Tables

**Table 1:** Identification and quantification (μg g^−1^±s.e.) of soluble arsenic species in cell fractions.

**As species**	***F***_**SC**_	***F***_**ENTO**_	***F***_EC_*	***F***_**BAC**_
As(III)	0 (±0)	0 (±0)	7.9 (±2.8)	0.6 (±1.3)
As(V)	892 (±284)†	4,498 (±983)‡	4,301 (±1,228)†	747 (±549)
MMAs	9.5 (±3.4)	78.9 (±14.1)	42.6 (±11.3)	9.8 (±5.1)
DMAs	0.2 (±0.2)	0 (±0)	2.4 (±1.7)	0.8 (±0.8)
AsB	1.4 (±0.4)	0 (±0)	136 (±12)	2.6 (±5.3)

ANOVA, analysis of variance; AsB, arsenobetaine; As(III), arsenite; As(V), arsenate; DMA, dimethylarsinic acid; *F*_BAC_, bacteria-enriched fraction; *F*_EC_, *Entotheonella* sp. and Cyanobacteria-enriched fraction; *F*_ENTO_, *Entotheonella* sp.-enriched fraction; *F*_SC_, sponge cell-enriched fraction; HSD, honest significant difference; MMA, monomethylarsonic acid.

^*^F_EC_ was excluded from among permuted ANOVA analysis, failing the independency assumption. *F*_EC_ was tested only for within difference in As species.

^†^Denotes significant difference for arsenic species tested within a given cell fraction. Testing was conducted by permuted ANOVA test and Tukey's HSD *post hoc* grouping.

^‡^Denotes significant difference for arsenic species tested among the cell fractions. Testing was conducted by permuted ANOVA test and Tukey's HSD *post hoc* grouping.

## References

[b1] Oremland R. S. & Stolz J. F. The ecology of arsenic. Science 300, 939–944 (2003). 12738852 10.1126/science.1081903

[b2] Dehairs F., Chesselet R. & Jedwab J. Discrete suspended particles of barite and the barium cycle in the open ocean. Earth Planet. Sci. Lett. 49, 528–550 (1980).

[b3] Neff J. M. Ecotoxicology of arsenic in the marine environment. Environ. Toxicol. Chem. 16, 917–927 (1997).

[b4] Menzie C. A., Southworth B., Stephenson G. & Feisthauer N. The importance of understanding the chemical form of a metal in the environment: the case of barium sulfate (barite). Hum. Ecol. Risk Assess. 14, 974–991 (2008).

[b5] Slyemi D. & Bonnefoy V. How prokaryotes deal with arsenic. Environ. Microbiol. Rep. 4, 571–586 (2012). 23760928 10.1111/j.1758-2229.2011.00300.x

[b6] Chow T. J. & Goldberg E. D. On the marine geochemistry of barium. Geochim. Cosmochim. Acta 20, 192–198 (1960).

[b7] Rainbow P. S. Trace metal concentrations in aquatic invertebrates: why and so what? Environ. Pollut. 120, 497–507 (2002). 12442773 10.1016/s0269-7491(02)00238-5

[b8] Taylor M. W., Radax R., Steger D. & Wagner M. Sponge-associated microorganisms: evolution, ecology, and biotechnological potential. Microbiol. Mol. Biol. Rev. 71, 295–347 (2007). 17554047 10.1128/MMBR.00040-06 PMC1899876

[b9] Gili J.-M. & Coma R. Benthic suspension feeders: their paramount role in littoral marine food webs. Trends Ecol. Evol. (Personal edition) 13, 316–321 (1998). 10.1016/s0169-5347(98)01365-2 21238320

[b10] De Goeij J. M. . Surviving in a marine desert: the sponge loop retains resources within coral reefs. Science 342, 108–110 (2013). 24092742 10.1126/science.1241981

[b11] Ribes M., Coma R., Atkinson M. J. & Kinzie R. A. Sponges and ascidians control removal of particulate organic nitrogen from coral reef water. Limnol. Oceanogr. 50, 1480–1489 (2005).

[b12] Maldonado M. . Siliceous sponges as a silicon sink: an overlooked aspect of benthopelagic coupling in the marine silicon cycle. Limnol. Oceanogr. 50, 799–809 (2005).

[b13] Weisz J. B., Lindquist N. & Martens C. S. Do associated microbial abundances impact marine demosponge pumping rates and tissue densities? Oecologia 155, 367–376 (2008). 18030495 10.1007/s00442-007-0910-0

[b14] Mayzel B., Aizenberg J. & Ilan M. The elemental composition of demospongiae from the Red Sea, Gulf of Aqaba. PLoS ONE 9, e95775 (2014). 24759635 10.1371/journal.pone.0095775 PMC3997428

[b15] Cebrian E., Uriz M.-J. & Turon X. Sponges as biomonitors of heavy metals in spatial and temporal surveys in northwestern mediterranean: multispecies comparison. Environ. Toxicol. Chem. 26, 2430–2439 (2007). 17941749 10.1897/07-292.1

[b16] Pan K., Lee O. O., Qian P. Y. & Wang W. X. Sponges and sediments as monitoring tools of metal contamination in the eastern coast of the Red Sea, Saudi Arabia. Mar. Pollut. Bull. 62, 1140–1146 (2011). 21453933 10.1016/j.marpolbul.2011.02.043

[b17] Patel B., Balani M. C. & Patel S. Sponge ‘sentinel' of heavy metals. Sci. Total Environ. 41, 143–152 (1985). 3983628 10.1016/0048-9697(85)90184-6

[b18] Keren R., Lavy A., Mayzel B. & Ilan M. Culturable associated-bacteria of the sponge *Theonella swinhoei* show tolerance to high arsenic concentrations. Front. Microbiol. 6, 154 (2015). 25762993 10.3389/fmicb.2015.00154 PMC4340220

[b19] Keren R., Lavy A. & Ilan M. Increasing the Richness of culturable arsenic-tolerant bacteria from *Theonella swinhoei* by addition of sponge skeleton to the growth medium. Microb. Ecol. 71, 873–886 (2016). 26809776 10.1007/s00248-015-0726-0

[b20] Ilan M., Gugel J. & Van Soest R. W. M. Taxonomy, reproduction and ecology of new and known Red Sea sponges. Sarsia 89, 388–410 (2004).

[b21] Magnino G., Sara A., Lancioni T. & Gaino E. Endobionts of the coral reef sponge *Theonella swinhoei* (Porifera, Demospongiae). Inverteb. Biol. 118, 213–220 (1999).

[b22] Wilson M. C. . An environmental bacterial taxon with a large and distinct metabolic repertoire. Nature 506, 58–62 (2014). 24476823 10.1038/nature12959

[b23] Schmidt E. W., Obraztsova A. Y., Davidson S. K., Faulkner D. J. & Haygood M. G. Identification of the antifungal peptide-containing symbiont of the marine sponge *Theonella swinhoei* as a novel delta-proteobacterium, ‘Candidatus *Entotheonella palauensis*'. Mar. Biol. 136, 969–977 (2000).

[b24] Phillips D. J. H. & Rainbow P. S. Strategies of trace metal sequestration in aquatic organisms. Mar. Environ. Res. 28, 207–210 (1989).

[b25] Yahel G., Sharp J. H., Marie D., Hase C. & Genin A. *In situ* feeding and element removal in the symbiont-bearing sponge *Theonella swinhoei*: bulk DOC is the major source for carbon. Limnol. Oceanogr. 48, 141–149 (2003).

[b26] Bazylinski D. A. & Frankel R. B. in Biomineralization Reviews in Mineralogy and Geochemistry Vol. 54, eds Dove P. M., DeYoreo J. J., Weiner S. 217–247Mineralogical Soc America (2003).

[b27] Frankel R. B. & Bazylinski D. A. Biologically induced mineralization by bacteria. Rev. Mineral. Geochem. 54, 95–114 (2003).

[b28] Edwards K. J. & Bazylinski D. A. Intracellular minerals and metal deposits in prokaryotes. Geobiology 6, 309–317 (2008). 18452538 10.1111/j.1472-4669.2008.00156.x

[b29] Konhauser K. O. Diversity of bacterial iron mineralization. Earth Sci. Rev. 43, 91–121 (1998).

[b30] Peng X. T. . Intracellular and extracellular mineralization of a microbial community in the Edmond deep-sea vent field environment. Sediment. Geol. 229, 193–206 (2010).

[b31] Rietveld H. M. A profile refinement method for nuclear and magnetic structures. J. Appl. Crystallogr. 2, 65–6 (1969).

[b32] Smith P. G. . X-ray absorption near-edge structure analysis of arsenic species for application to biological environmental samples. Environ. Sci. Technol. 39, 248–254 (2005). 15667101 10.1021/es049358b

[b33] Arai Y., Elzinga E. J. & Sparks D. L. X-ray absorption spectroscopic investigation of arsenite and arsenate adsorption at the aluminum oxide-water interface. J. Colloid Interface Sci. 235, 80–88 (2001). 11237445 10.1006/jcis.2000.7249

[b34] Hohmann C. . Molecular-level modes of As binding to Fe(III) (oxyhydr)oxides precipitated by the anaerobic nitrate-reducing Fe(II)-oxidizing *Acidovorax* sp. strain BoFeN1. Geochim. Cosmochim. Acta 75, 4699–4712 (2011).

[b35] Finch A. A., Allison N., Steaggles H., Wood C. V. & Mosselmans J. F. W. Ba XAFS in Ba-rich standard minerals and the potential for determining Ba structural state in calcium carbonate. Chem. Geol. 270, 179–185 (2010).

[b36] Escalante G. . Arsenic resistant bacteria isolated from arsenic contaminated river in the Atacama Desert (Chile). Bull. Environ. Contam. Toxicol. 83, 657–661 (2009). 19779656 10.1007/s00128-009-9868-4

[b37] Stolz J. F., Basu P., Santini J. M. & Oremland R. S. Arsenic and selenium in microbial metabolism*. Annu. Rev. Microbiol. 60, 107–130 (2006). 16704340 10.1146/annurev.micro.60.080805.142053

[b38] Weiner S. & Dove P. M. in Biomineralization Reviews in Mineralogy and Geochemistry Vol. 54, eds Dove P. M., DeYoreo J. J., Weiner S. 1–29Mineralogical Society of America (2003).

[b39] Chávez-Capilla T., Maher W., Kelly T. & Foster S Evaluation of the ability of arsenic species to traverse cell membranes by simple diffusion using octanol–water and liposome–water partition coefficients. J. Environ. Sci. 49, 222–232 (2016). 10.1016/j.jes.2016.08.007 29216971

[b40] Morth J. P. & Perdreau H. in Encyclopedia of Metalloproteins eds Kretsinger R. H., Uversky V. N., Permyakov E. A. 241–244Springer New York (2013).

[b41] Erez J. in *Biomineralization Reviews in Mineralogy & Geochemistry* Vol. 54 (eds Dove, P. M., DeYoreo, J. J. & Weiner, S.) 115–149Mineralogical Society of America (2003).

[b42] Lonhienne T. G. A. . Endocytosis-like protein uptake in the bacterium *Gemmata obscuriglobus*. Proc. Natl Acad. Sci. USA 107, 12883–12888 (2010). 20566852 10.1073/pnas.1001085107 PMC2919973

[b43] Yamaoka Y., Carmona M. L., Oclarit J. M., Jin K. & Shibata Y. Characterization of water-soluble organoarsenic compounds in marine sponges. Appl. Organomet. Chem. 20, 545–548 (2006).

[b44] Banerjee S., Datta S., Chattyopadhyay D. & Sarkar P. Arsenic accumulating and transforming bacteria isolated from contaminated soil for potential use in bioremediation. J. Environ. Sci. Health Part A 46, 1736–1747 (2011). 10.1080/10934529.2011.623995 22175878

[b45] Yin X. X., Wang L. H., Bai R., Huang H. & Sun G. X. Accumulation and transformation of arsenic in the blue-green alga *Synechocysis* sp PCC6803. Water Air Soil Pollut. 223, 1183–1190 (2012).

[b46] Basu A., Saha D., Saha R., Ghosh T. & Saha B. A review on sources, toxicity and remediation technologies for removing arsenic from drinking water. Res. Chem. Intermed. 40, 447–485 (2014).

[b47] Newman D. K., Beveridge T. J. & Morel F. Precipitation of arsenic trisulfide by *Desulfotomaculum auripigmentum*. Appl. Environ. Microbiol. 63, 2022–2028 (1997). 16535611 10.1128/aem.63.5.2022-2028.1997 PMC1389166

[b48] Saier M. H. Jr & Bogdanov M. V. Membranous organelles in bacteria. J. Mol. Microbiol. Biotechnol. 23, 5–12 (2013). 23615191 10.1159/000346496

[b49] Bewley C. A., Holland N. D. & Faulkner D. J. Two classes of metabolites from *Theonella swinhoei* are localized in distinct populations of bacterial symbionts. Experientia 52, 716–722 (1996). 8698116 10.1007/BF01925581

[b50] de Chaumont F. . Icy: an open bioimage informatics platform for extended reproducible research. Nat. Meth. 9, 690–696 (2012). 10.1038/nmeth.2075 22743774

[b51] EPA. The SW-846 C\compendium method 3050B: acid digestion of sediments, sludges, and soils, revision 2 (1996).

[b52] Cornelia H. & Britta P.-F. Thioarsenate transformation by filamentous microbial mats thriving in an alkaline, sulfidic hot spring. Environ. Sci. Technol. 46, 4348–4356 (2012). 22380721 10.1021/es204277j

[b53] Herskovits A. A., Shimoni E., Minsky A. & Bibi E. Accumulation of endoplasmic membranes and novel membrane-bound ribosome-signal recognition particle receptor complexes in Escherichia coli. J. Cell Biol. 159, 403–410 (2002). 12417577 10.1083/jcb.200204144 PMC2173083

[b54] Fitch A. N. The high resolution powder diffraction beam line at ESRF. J. Res. Natl Inst. Stand. Technol. 109, 133–142 (2004). 27366602 10.6028/jres.109.010 PMC4849623

[b55] Toby B. H. GSAS-II: the genesis of a modern open-source all purpose crystallography software package. J. Appl. Crystallogr. 46, 544 (2013).

[b56] Wolf S. G., Houben L. & Elbaum M. Cryo-scanning transmission electron tomography of vitrified cells. Nat. Methods 11, 423 (2014). 24531421 10.1038/nmeth.2842

[b57] Marcus M. A. . Beamline 10.3. 2 at ALS: a hard X-ray microprobe for environmental and materials sciences. J. Synchrotron Radiat. 11, 239–247 (2004). 15103110 10.1107/S0909049504005837

[b58] Fakra S. C. . Correlative cryogenic spectro-microscopy to investigate Selenium bioreduction products. Environ. Sci. Technol. doi:10.1021/acs.est.5b01409 (2015). 10.1021/acs.est.5b01409 26371540

[b59] Kelly S., Hesterberg D. & Ravel B. Analysis of soils and minerals using X-ray absorption spectroscopy. Methods Soil Anal Part 5, 387–463 (2008).

[b60] Castillo-Michel H. . Localization and speciation of arsenic in soil and desert plant Parkinsonia florida using μXRF and μXANES. Environ. Sci. Technol. 45, 7848–7854 (2011). 21842861 10.1021/es200632s PMC3185050

[b61] Hammersley A., Svensson S., Hanfland M., Fitch A. & Hausermann D. Two-dimensional detector software: from real detector to idealised image or two-theta scan. Int. J. High Pressure Res. 14, 235–248 (1996).

[b62] Fuller M. E. . Development of a vital fluorescent staining method for monitoring bacterial transport in subsurface environments. Appl. Environ. Microbiol. 66, 4486–4496 (2000). 11010903 10.1128/aem.66.10.4486-4496.2000 PMC92329

[b63] R Foundation for Statistical Computing. R: A Language and Environment for Statistical Computing V. 2.15.1 R Foundation for Statistical Computing (2012).

[b64] R Development Core Team. A language and environment for statistical computing. R Foundation for Statistical Computing Vienna, Austria (2008) http://www.R-project.org.

