# Supplementary material for: Sponge-associated bacteria mineralize arsenic and barium on intracellular vesicles
Source: Nat Commun. 2017 Feb 24;8:14393. doi: 10.1038/ncomms14393 (PMC5333131; doi:10.1038/ncomms14393)
Supplement: Supplementary Information — Supplementary Tables and Supplementary Figures [file ncomms14393-s1.pdf]

Supplementary Table 1 Concentration of arsenic and barium (μg g^{-1} ±SE) in cell enriched fractions

**Cell fraction**	**Total As**	**Total Ba**
F_{SC}	1302 (±371)^{b*}	11434 (±2923)^{ab}
F_{ENTO}	12072 (±3743)^{a}	26420 (±6451)^{a}
F_{BAC}	1313 (±1523)^{b}	10160 (±1752)^{b}
F_{EC}**	12142 (±3406)	16118 (±3918)

*Superscript letters denote Tukey's HSD *post-hoc* grouping after permuted ANOVA. Arsenic- F= 7.856, p= 0.00657. Barium- F= 4.509, p= 0.0346.

** F_{EC} was excluded from permuted ANOVA analysis, failing the independency assumption. F_{ENTO} and F_{EC} statistical comparison is presented in Table 2

Supplementary Table 2 Comparison of element and species concentration (μg g^{-1}), in cell fractions containing *Entotheonella* sp.

**Measurement**	F_{ENTO}	F_{EC}
Total As	12072.0	12142.0
Total Ba	26420.0	16118.0
Total S	4326.0	11038.0*
Total P	3364.0	5314.0*
Soluble As	4576.9	4490.3
Soluble Ba	541.4	670.2
Soluble S	912.4	5394.0*
Soluble P	641.4	1029.8*
AsIII	0.0	7.9
AsV	4498.0	4301.0
MMA	78.9	42.6
DMA	0.0	2.4
AsB	0.0	136.4*

* denotes significant difference between samples according to exact permutation test estimated by 999 Monte Carlo replications

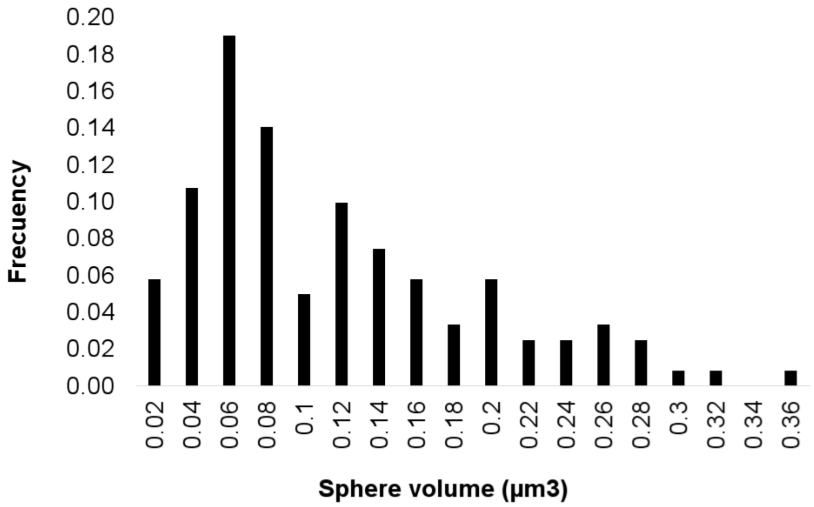

Supplementary Figure 1 Histogram of sphere volume (µm^3). Volume of spheres was binned at intervals of 0.02 µm^3. n=121, range 0.01-0.35 µm^3.

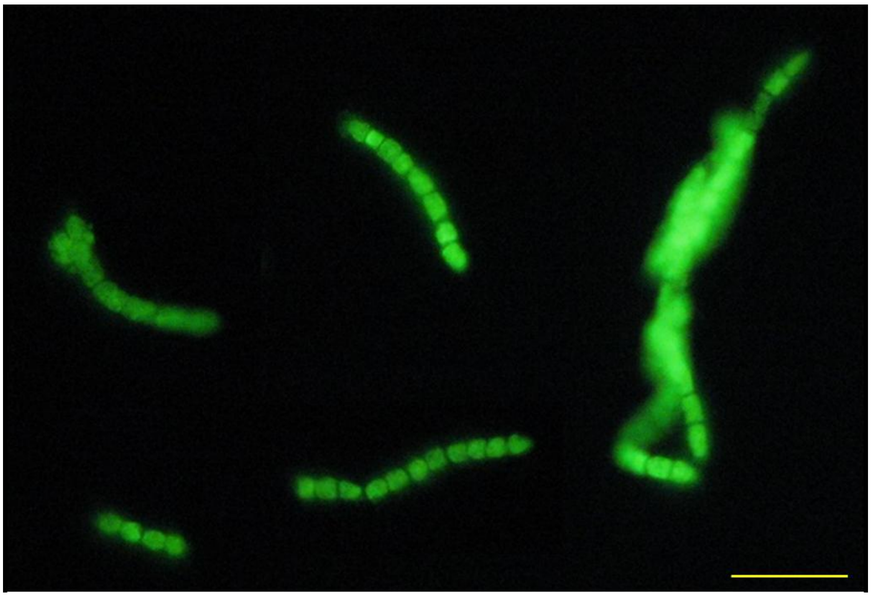

Supplementary Figure 2 Viability of Entotheonella sp. Epifluorescence microscope image of filaments stained with vital stain CFDA/SE (ex- 492 nm, em- 517 nm). All filaments and all cell units are stained, confirming vitality. Scale bar 20 µm.

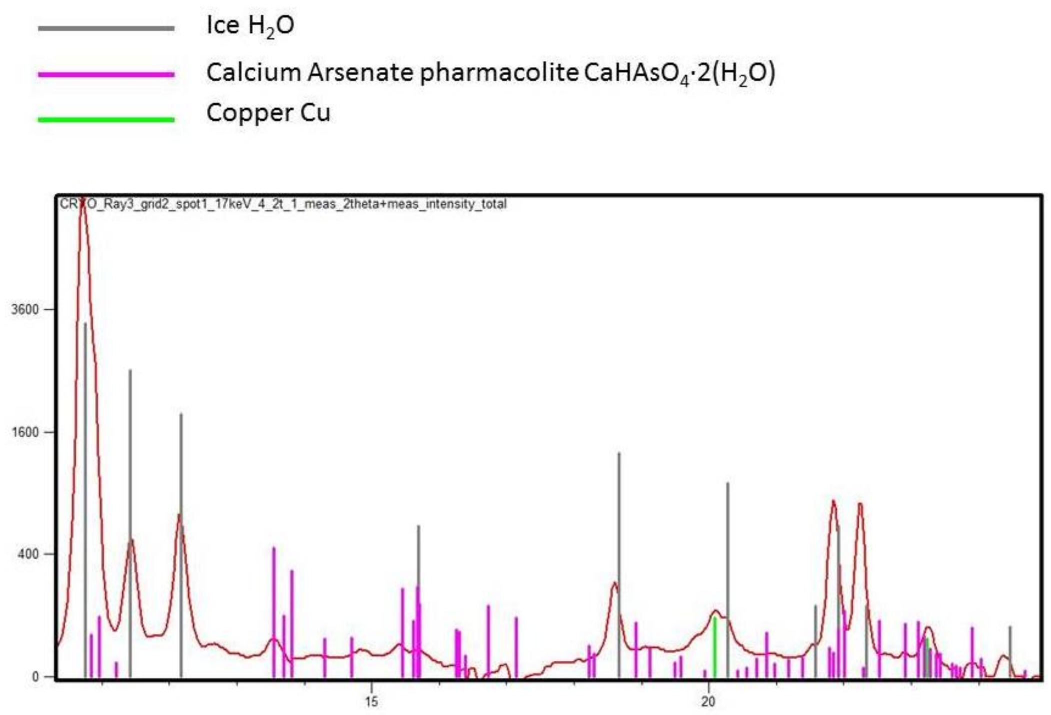

Supplementary Figure 3 Micro-X-ray diffraction profile of Entotheonella sp filament taken at 17 keV for 240 sec., showing the presence of crystalline pharmacolite. The Cu signal originates from the Cu grid.

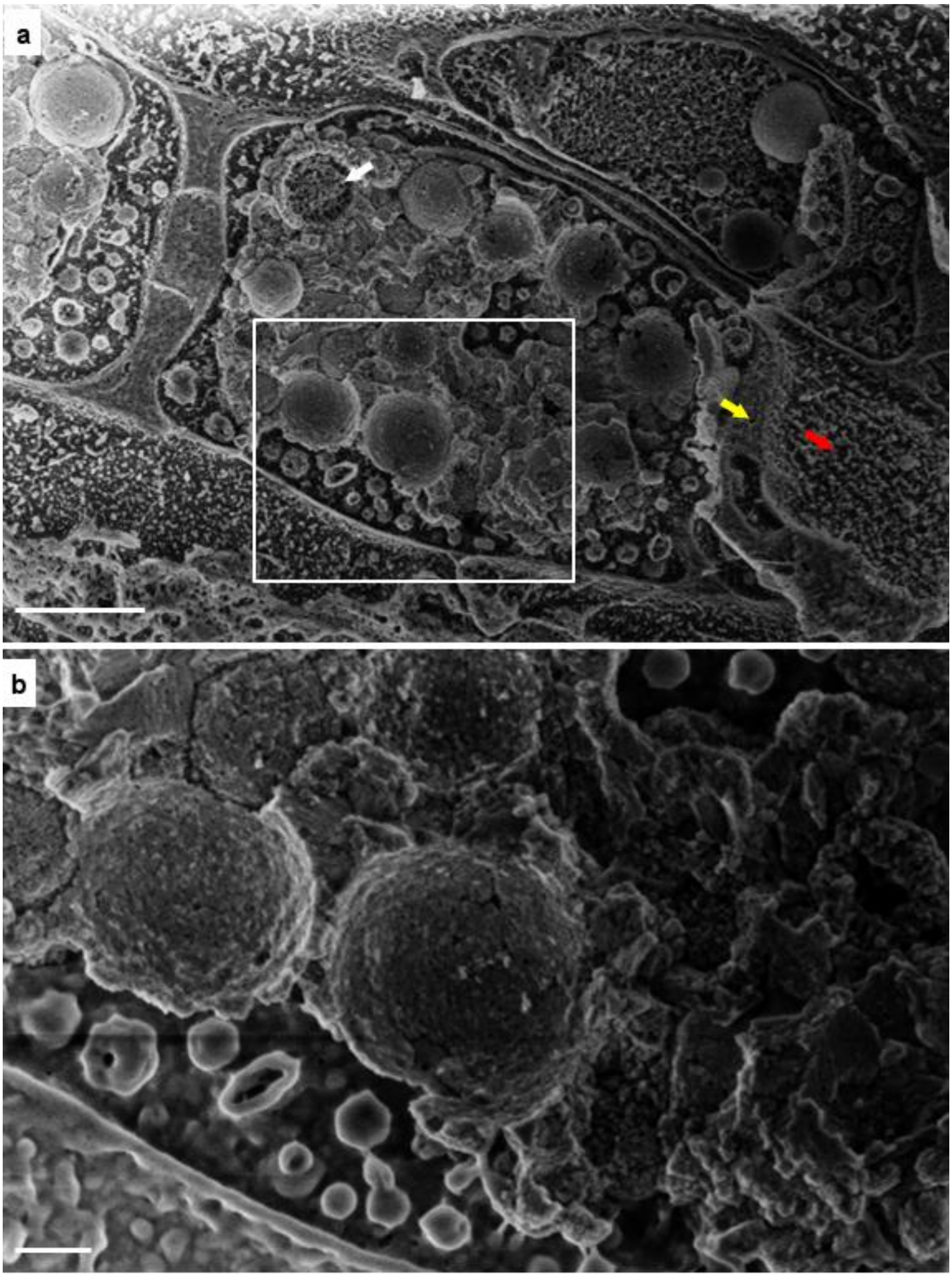

Supplementary figure 4 merged mineralized spheres in *Entotheonella* sp. a, Cryo-SEM micrograph of freeze fractured *Entotheonella* sp. filament. Scale bar 1 µm. On the right hand side of the image the fracture runs through the outer sheaf (red arrow) and outer membrane (yellow arrow). In the inner space of the cell unit, all spheres are merged. The shape of the individual spheres is still discernible but there is additional mineralization between the spheres linking them all together. On the upper left side of the cell, the fracture runs through the sphere (white arrow). The inner membrane is absent from the cell (as is the case with all such observations). The edge of the mineral (white square) was further examined under higher magnification, b, and the absence of the inner membrane yet again confirmed. Scale bar 200 nm.
